# Bioequivalence of twice-daily oral tacrolimus in transplant recipients: More evidence for consensus?

**DOI:** 10.1371/journal.pmed.1002429

**Published:** 2017-11-14

**Authors:** Simon Ball

**Affiliations:** 1 Department of Nephrology, Queen Elizabeth Hospital Birmingham, Birmingham, United Kingdom; 2 Institute of Immunology and Immunotherapy, College of Medical and Dental Sciences, University of Birmingham, Birmingham, United Kingdom

## Abstract

In this Perspective on the clinical trial by Rita Alloway and colleagues, Simon Ball explains the benefits to healthcare systems and individual patients of the bioequivalence established between generic and brand-name formulations of an immunosuppressive drug in transplant recipients.

In many healthcare systems, the high cost of long-term immunosuppression is borne by the transplant recipient. Even in the world’s wealthiest economies the annual cost of an individual’s immunosuppression is a significant proportion of median income. This can contribute to nonadherence with the prescribed immunosuppressive regime and therefore economically driven health inequality, even in the most highly developed healthcare systems [1]. In developing countries, access to immunosuppression not only affects outcome but also the very opportunity to undergo transplantation [2]. The careful use of cheaper, generic formulations of immunosuppression therefore has the potential to significantly benefit healthcare systems by reducing the long-term cost of immunosuppression and improve patient-level outcomes by promoting long-term adherence and access to transplantation. In this issue of *PLOS Medicine*, Alloway and colleagues have addressed an important question regarding the bioequivalence of different formulations of tacrolimus in transplant recipients. Bioequivalence is a prerequisite for the extrapolation of clinical outcome study results using the originator’s formulation to the use of generic formulations [3].

The calcineurin inhibitor tacrolimus was approved by the United States Food and Drug Administration (FDA) for immunosuppression of liver transplant recipients in 1994 and kidney transplant recipients in 1997. By 2007, it was administered to more than 80% of kidney transplant recipients in the US Renal Data System. In that year the ELiTE-SYMPHONY study was published comparing 4 different immunosuppressive regimes in kidney transplantation [4]. Its findings contributed to FDA approval in 2009 for the combined use of mycophenolate mofetil and tacrolimus, which has become the standard of therapy for kidney transplantation in many healthcare systems. In that year, the first generic formulation of tacrolimus became available, and there are currently 6 products approved by the FDA.

Tacrolimus has a narrow therapeutic index and is subject to routine therapeutic drug monitoring and dose adjustment. High peripheral blood concentrations of tacrolimus are associated with the nonspecific consequences of over-immunosuppression and specific effects of calcineurin inhibition, notably nephrotoxicity, neurotoxicity, and thrombotic microangiopathy. Low peripheral blood concentrations of tacrolimus are associated with acute rejection. In kidney transplant recipients, there is also evidence that high within-patient variability in trough tacrolimus concentration is associated with adverse outcomes, including acute rejection, chronic antibody mediated rejection, and ultimately allograft failure [5]. This association may in part be consequent upon nonadherence with the prescribed immunosuppression regime, but other contributory factors are also likely to be important [6].

Given a narrow therapeutic index and the clinical significance of high within-patient variability in drug exposure, there has been some reluctance amongst clinicians and patients to consider changing tacrolimus formulation [7]. Patient and professional groups have published advice that immunosuppressive agents should be prescribed according to brand, have expressed concerns over the use of generic formulations, and have made recommendations on the utilization of the branded and generic formulations [8]. This is partly based upon the fact that FDA approval requires an average bioequivalence confidence interval of 80%–125%, established in healthy individuals. Arguably, this is inadequate for a medication with a narrow therapeutic index such as tacrolimus, in which short periods of inadequate drug exposure may be sufficient to release alloimmune mechanisms, irreversibly. The acceptance interval for the area under the concentration curve to demonstrate bioequivalence has therefore been reduced for narrow therapeutic index drugs by the European Medicines Agency (EMA) (90%–111.1%) and Health Canada (90%–112%).

In response to the concerns of patients and professionals and a resulting call for investigator-led studies by the FDA, Alloway and colleagues have undertaken a detailed prospective, pharmacokinetic study in kidney and liver transplant recipients [3]. The originator’s product, Prograf, has been compared with 2 generic formulations in a 3-treatment, 6-period crossover design. The study therefore considers within-subject variability in drug exposure over time and across products. In addition to conventional bioequivalence, scaled average bioequivalence (SCABE) can then be reported. SCABE is a statistical method that accounts for the degree of within-patient variability in reference drug exposure when assessing bioequivalence. The 2 generic formulations studied were chosen as those exhibiting the greatest difference in pharmacokinetic parameters, based upon studies in a healthy population. Though, in this study, both ‘Generic Lo’ and ‘Generic Hi’ products tended towards greater drug exposure than did Prograf. The SCABE for area under the concentration curve for the ‘Generic Lo’ product exhibited upper confidence limits of 111.3% for kidney and 112.1% for liver transplant recipients, only just above the EMA definitions of bioequivalence. Other criteria for bioequivalence of generic and originator products were met in this population of transplant recipients. The known high level of inter-patient variability in drug metabolism was reiterated in this study.

These data suggest that the use of generic formulations of twice-daily tacrolimus in adults with stable allograft function is likely to be safe. However, it is notable that only 7 of the 71 patients studied were African American. This may be relevant since the African American population exhibits different pharmacokinetics and within-patient variability compared with the white population [9], and this warrants ongoing vigilance. The overall conclusion, though, is strongly in favor of the operational equivalence of these different formulations. That is not to say that formulations should be dispensed entirely interchangeably, since a consistent preparation is likely to promote patient understanding, involvement in medicines management, and regime adherence [10]. A consideration that is true irrespective of drug and formulation but is particularly important for some classes of therapy, such as transplant immunosuppression and anti-retroviral therapy [11], in which even low-level nonadherence has potentially irreversible consequences for disease progression. A change in tacrolimus formulation that is effectively communicated to the transplant recipient or the primary use of a particular formulation can therefore be reasonably undertaken, based upon knowledge derived from the originator product.

The study by Alloway and colleagues is a significant achievement, providing good evidence of bioequivalence between twice-daily formulations of tacrolimus. It accounts for within-patient variability in drug exposure over time in ways that have not previously been adequately dealt with in the literature. Given the importance of consistent dosing with optimal immunosuppression and the well-documented economic barriers to doing so in different settings, this high level of assurance for the use of generic formulations will not only improve value derived across healthcare systems but, crucially, also improve individual patient-level outcomes in the long-term and patient access to treatment across a range of healthcare economies.

## Abbreviations

EMA: European Medicines Agency
FDA: United States Food and Drug Administration
SCABE: scaled average bioequivalence
